# ﻿Tetramoriumsinensis sp. nov., a parabiotic ant from China, with a key to the Tetramoriuminglebyi group (Hymenoptera, Formicidae)

**DOI:** 10.3897/zookeys.1236.137346

**Published:** 2025-04-25

**Authors:** Benan Zhang, Congcong Du, Zhilin Chen

**Affiliations:** 1 Key Laboratory of Ecology of Rare and Endangered Species and Environmental Protection (Guangxi Normal University), Ministry of Education, Guilin 541006, China Guangxi Normal University Guilin China; 2 Guangxi Key Laboratory of Rare and Endangered Animal Ecology, Guangxi Normal University, Guilin 541006, China Guangxi Normal University Guilin China

**Keywords:** Identification key, Myrmicinae, new species, parabiosis, taxonomy

## Abstract

In this paper, *Tetramoriumsinensis***sp. nov.**, a parabiotic ant, is described. It was discovered within the nest of the queenless ant *Diacammarugosum* (Le Guillou, 1842) in Fenghuang Mountain Park, Zhongshan, Guangdong Province, China. Additionally, a key to the *Tetramoriuminglebyi* group based on the worker caste is provided.

## ﻿Introduction

Social Hymenoptera, including bees, wasps, and ants, exhibit a wide range of social behaviors and nesting habits. Among these are cases in which more than one species can be found in one nest. This phenomenon can be categorized into two primary types: mixed colonies and compound nests. Mixed colonies are a phenomenon where different species or populations of animals live and breed together in the same habitat, such as in social parasitism (Rabeling 2021). Compound nests are structures composed of multiple nests, which may belong to the same species or different species; the types have been classified as plesiobiosis, cleptobiosis, lestobiosis, xenobiosis, and parabiosis (Hölldobler and Wilson 1990).

The ant genus *Tetramorium* belongs to the subfamily Myrmicinae (Hymenoptera, Formicidae), and was initially proposed by Mayr in 1855, with *Formicacaespitum* (Roger, 1862) designated as the type species through subsequent designation by Girard in 1879. Since that time, the classification of the various species within *Tetramorium* has become increasingly complex, with numerous junior and senior synonyms complicating the identification process (Bolton 2025).

The genus *Tetramorium* is the fourth largest within the Myrmicinae subfamily of Formicidae, encompassing 603 species that are extensively distributed across diverse biogeographic regions; notably, it lacks endemic species in the Neotropical region and is represented entirely by introduced species there (Bolton 2025). Taxonomic revision of this genus has been primarily conducted in specific regions (Bolton 1976, 1977, 1979, 1980; Radchenko 1992; Hita Garcia and Fisher 2014), and involves only certain species groups (Hita Garcia et al. 2010; Agavekar et al. 2017). Currently, *Tetramorium* is divided into 53 species groups, with most species featuring small eyes belonging to the *Tetramoriumshiloense* group and the *Tetramoriuminglebyi* group.

The *Tetramoriuminglebyi* species group was initially recognized by Agavekar et al. (2017). This is a small species group, comprising only five species, all of which are from India. The main characteristics are small eyes and a strongly concave base of the first gastral tergite in dorsal view, which are crucial for distinguishing it from other species. The most significant characteristic of *Tetramoriumsinensis* sp. nov. is its small eyes, which clearly distinguish it from other *Tetramorium* species in China. Due to the small eyes of the new species, we describe this new species as a member of the *T.inglebyi* group. A key to the *T.inglebyi* group based on the worker caste is provided.

## ﻿Material and methods

Twenty-five specimens of *Tetramoriumsinensis* sp. nov. were collected from Fenghuang Mountain Park, Shaxi Town, Zhongshan City, Guangdong Province, China. The type specimens of this new species have been deposited in the following repositories: (1) **GXNU** (Insect Collection, Guangxi Normal University, Guilin, Guangxi, China), (2) **SWFU** (Insect Collection, Southwest Forestry University, Kunming, Yunnan Province, China), and (3) **IZCAS** (Institute of Zoology, Chinese Academy of Sciences, Beijing, China). Morphological observations and identifications were conducted using a Nikon SMZ745 stereoscopic microscope, and photographs were taken and measurements obtained using a KEYENCE ultra-Depth of Field three-dimensional microscopy system (VHX-6000). The type specimen images of four species have been made available on the AntWeb (http://www.antweb.org) and *T.triangulatum* is available on the AntWiki (http://www.antwiki.org).

The measurement standard in this paper adheres to the definition provided by Hita Garcia and Fisher (2014). The unit of measurement is millimeters (mm), and the relevant measurement abbreviations are as follows:

**ED** (Eye diameter): Maximum diameter of eyes.

**HL** (Head length): Head in full-face view, the length from the midpoint of the anterior clypeal margin to the midpoint of the posterior margin of the head (when the midpoint of the anterior clypeal margin or the posterior margin of the head is depressed, the Central Line of the protruding part on both sides shall prevail).

**HW** (Head width): Head in full-face view, the maximum width of the head (excluding eyes).

**ML** (Mesosoma length): Mesosoma diagonal length (from the junction of the pronotum and neck to the lower end of the metapleural lobe).

**PH** (Pronotal height): Body in lateral view, the maximum height of the prosternum.

**PW** (Pronotal width): Body in dorsal view, the maximum width of the prosternum (excluding spines or denticles).

**PTL** (Petiolar node length): Maximum length of petiolar (in dorsal view).

**PTH** (Petiolar node height): Maximum height of the petiolar, excluding the subpetiolar process (in lateral view).

**PTW** (Petiolar node width): Maximum width of petiolar (in dorsal view).

**PPH** (Postpetiole height): In lateral view, the maximum height of the postpetiole.

**PPL** (Postpetiole length): In the dorsal view, the maximum length of the postpetiole.

**PPW** (Postpetiole width): In the dorsal view, the maximum width of the postpetiole.

**SL** (Scape length): Maximum length of antennal scape excluding globular base.

**CI** (Cephalic index): HW×100/HL

**DMI** (Dorsal mesosoma index): PW×100/ML

**DPeI** (Dorsal petiole index): PTW×100/PTL

**DPpI** (Dorsal postpetiole index): PPW×100/PPL

**LMI** (Lateral mesosoma index): PH×100/ML

**LPeI** (Lateral petiole index): PTL×100/PTH

**LPpI** (Lateral postpetiole index): PPL×100/PPH

**OI** (Ocular index): ED×100/HW

**PeNI** (Petiolar node index): PTW×100/PW

**PpNI** (Postpetiolar node index): PPW×100/PW

**PPI** (Postpetiole index): PPW×100/PTW

**SI** (Scape index): SL×100/HW

## ﻿Results

### ﻿List of the *Tetramoriuminglebyi*-group species

*T.elisabethae* Forel, 1904

*T.inglebyi* Forel, 1902

*T.jarawa* Agavekar, Hita Garcia & Economo, 2017

*T.myops* Bolton, 1977

*T.sinensis* sp. nov.

*T.triangulatum* Bharti & Kumar, 2012

### ﻿Description of new species

#### ﻿ Tetramorium sinensis sp. nov.

Taxon classificationAnimaliaHymenopteraFormicidae

562F9757-60E0-5B2D-B9B5-9669F67A9659

https://zoobank.org/420B95D7-C3EB-4FEF-A535-C343A263B327

Figs 1 , 2

##### Material examined.

***Holotype* worker**: China • Guangdong Province, Zhongshan City, Fenghuang Mountain Park; 22°29'18"N, 113°18'32"E; elev. 35 m; in *D.rugosum* nest; 08–November–2021, Huasheng Huang leg.; No. GXNU2102704; (GXNU: GXNU2102704). ***Paratype* worker**: China • 25 paratype workers from the same colony as the holotype (23 workers, GXNU; 1 worker, SWFU; 1 worker, IZCAS).

##### Diagnosis.

Head in full-face view subrectangular, slightly longer than broad, long longitudinally striate from the anterior clypeal to the middle of head, lateral and posterior part of head slightly reticulate; eyes small, with 3–4 ommatidia in the greatest diameter. Mesosoma in dorsal view longitudinally sculptured, pronotum front slightly reticulate; in lateral view, distinctly dense transverse sculptured, propodeal spines short triangular and the tip straight. propodeal lobe angular. Petiole in dorsal view circular, as long as broad.

##### Measurements and indices.

Holotype worker (*N = 25*): ***Measurements***: HL: 0.59–0.64; HW: 0.54–0.57; SL: 0.36–0.38; PH: 0.30–0.32; PW: 0.38–0.42; ML: 0.68–0.72; ED: 0.05–0.07; PTL: 0.20–0.21; PTH: 0.20–0.21; PTW: 0.19–0.20; PPH: 0.20–0.21; PPL: 0.17–0.19; PPW: 0.23–0.25. ***Indices***: CI: 89.06–91.53; SI: 66.67; OI: 9.26–12.28; DMI: 55.88–58.33; LMI: 44.12–44.44; PeNI: 47.62–50.00; LPeI: 100.00; DpeI: 95.00–95.24; PpNI: 59.52–60.53; LPpI: 85.00–90.48; DPpI: 131.58–135.29; PPI: 121.05–125.00.

##### Description.

**Figure 1.**
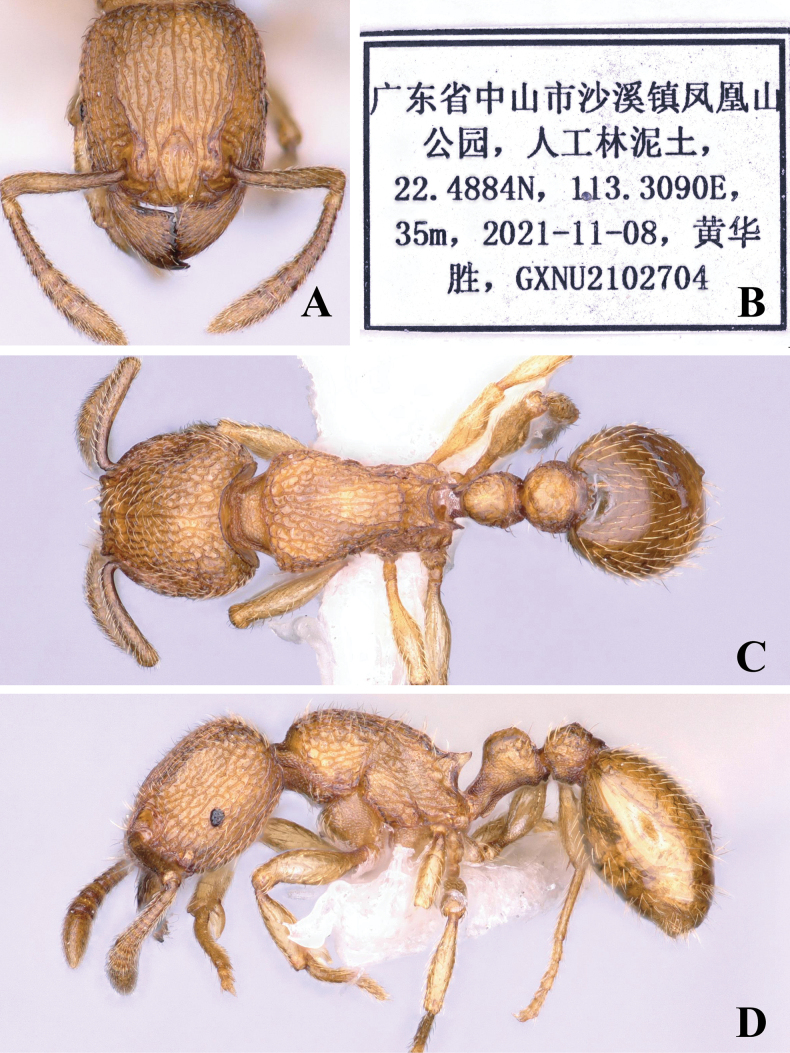
*Tetramoriumsinensis* sp. nov., worker. Head in full-face view (**A**), label of holotype (**B**), body in dorsal view (**C**), body in lateral view (**D**).

***Head*.** Antennae with 12 segments; antennal scape slightly curved; scape reaching two-thirds of the length of the head. Head in full-face view subrectangular, slightly longer than broad, lateral margins convex, posterior margin slightly concave in middle, with posterolateral corner rounded, frontal carina short, only reaching to the middle of head. Anterior clypeus nearly straight, antennal scrobe obvious (Fig. 1A); in lateral view, the diameter of the eye less than half of the maximum diameter of the enlarged part of the antennal terminal segment (Fig. 1D).

***Mesosoma*.** In dorsal view, lateral margins slightly convex, anterior margin convex (Fig. 1C); in lateral view, dorsal outline strongly convex, with transverse curve; promesonotal suture and metanotal groove inconspicuous; mesopleuron demarcated from pronotum by a distinct suture, but not demarcated from mesonotum and metapleuron; propodeal spines short triangular (Fig. 1D).

***Metasoma*.** In dorsal view, petiole circular, as long as broad; lateral margins slightly convex; anterior margin convex and posterior margin slightly concave (Fig. 1C); in lateral view, petiolar node slightly convex dorsal outline, slightly higher than long, with bilateral edge sloped slightly (Fig. 1D). Postpetiole in dorsal view clearly larger than petiole, oval, lateral margins apparently convex, as long as broad (Fig. 1C); in lateral view, slightly convex dorsal outline (Fig. 1D). In dorsal view, anterior margin of gaster obviously concave (Fig. 1C).

**Figure 2.**
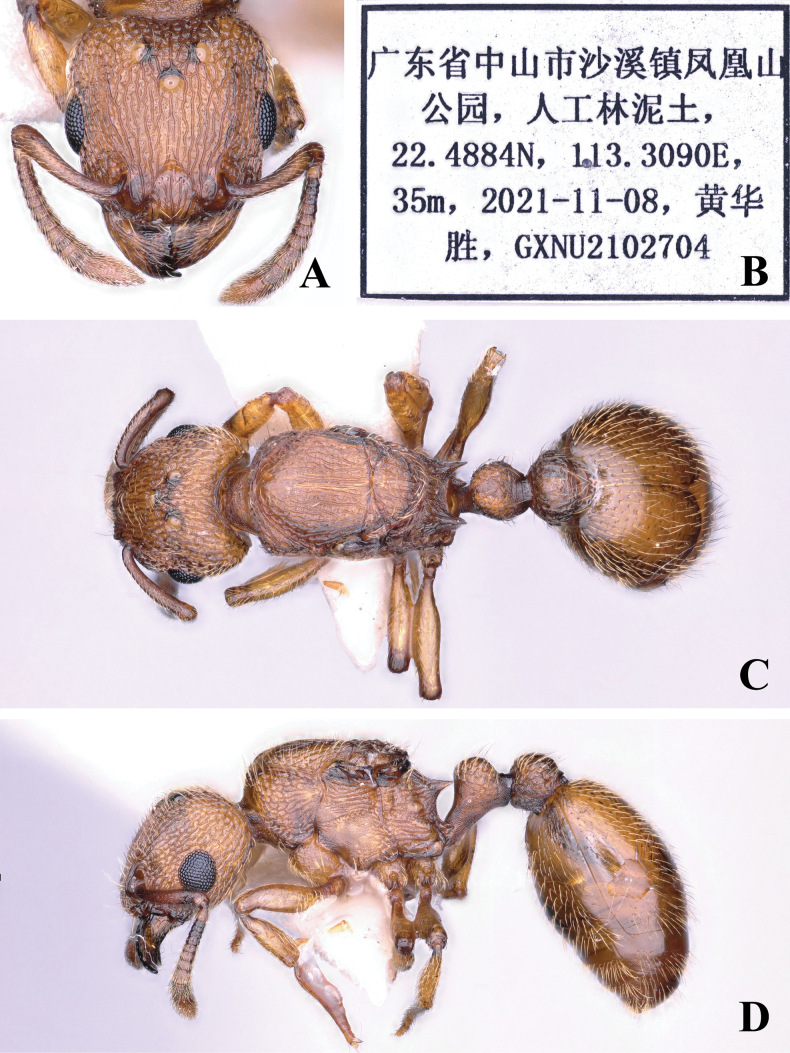
*Tetramoriumsinensis* sp. nov., queen. Head in full-face view (**A**), label of holotype (**B**), body in dorsal view (**C**), body in lateral view (**D**).

***Sculpture*.** Mandibles and clypeus longitudinally striate; antennal scape finely puncta; frontal area longitudinal striate, lateral and posterior part of head slightly reticulate (Fig. 1A). The pronotum reticulate, the mesonotum and metanotum longitudinally striate (Fig. 1C); the lateral sides of the mesosoma with transverse curve and sparsely puncta (Fig. 1D). Dorsum of petiole sparsely rugose (Fig. 1C). Coxa, peduncle, subpetiolar process with dense puncta (Fig. 1D). Gaster smooth and shining (Fig. 1C).

***Pilosity*.** Body entirely covered with abundant decumbent, sub-erect, and erect hairs (Fig. 1A, C, D).

***Coloration*.** Body brown. Antennae and legs slightly yellowish-brown (Fig. 1).

##### Etymology.

The new species name is derived from the Latin word “Sina” (sinensis), a reference to the type locality.

##### Distribution.

China (Guangdong).

##### Biology.

**Figure 3.**
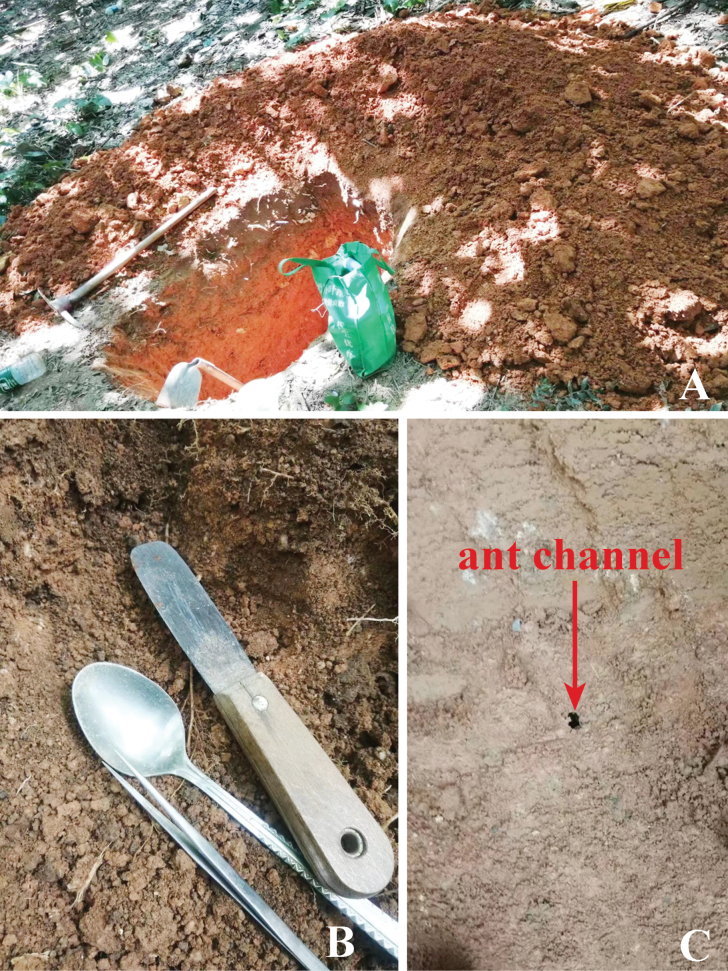
Habitat (**A**), collection tools (**B**), and ant channels (**C**).

**Figure 4.**
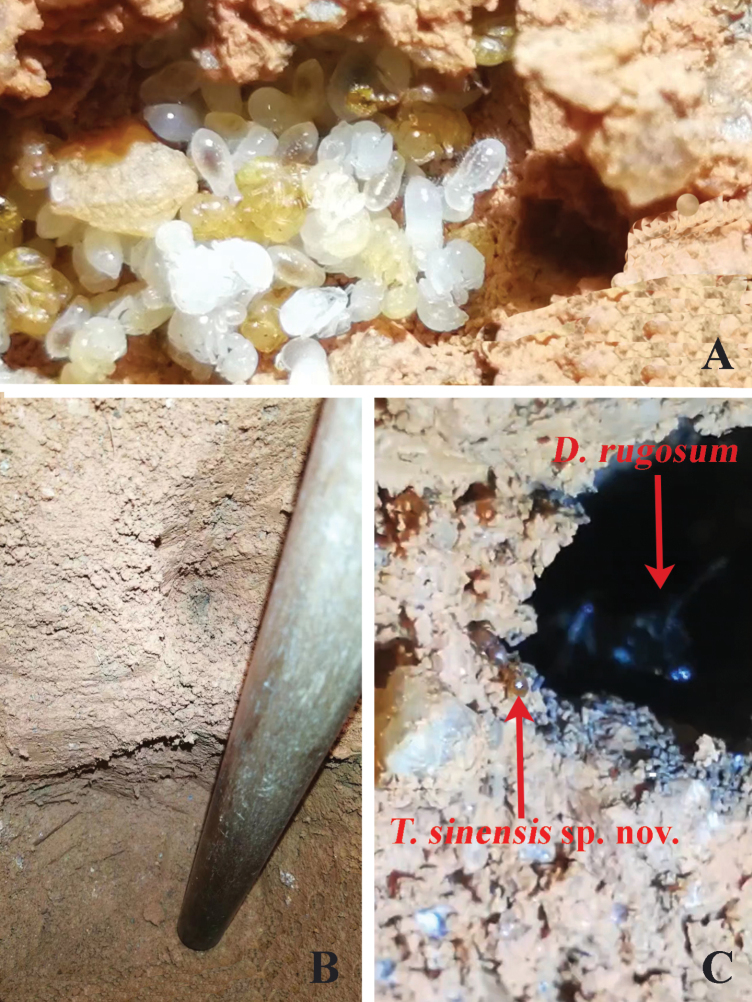
The nest of parabiotic ants (**A**), nest depth (**B**), *Tetramoriumsinensis* sp. nov. and *Diacammarugosum* (**C**).

The new species was collected multiple times from the nests of the queenless ant *Diacammarugosum* (Le Guillou, 1842) in the soil of a plantation forest in Fenghuang Mountain Park, Shaxi Town, Zhongshan City, Guangdong Province, China. Consequently, in order to test the relationship between them, a detailed excavation of one of the nests was carried out by Huasheng Huang (Fig. 3A). Employing a hoe and pick to ascertain the direction of the ant without causing damage to the ant path whenever possible (Fig. 3C), small tools like tweezers and spoons were then used to trace the ant path (Fig. 3B). After an intensive 6-hour excavation, Huasheng Huang discovered both *D.rugosum* and *T.sinensis* sp. nov. in the same nest area at a depth of 1.4 m (Fig. 4B, C). Once the shallow loose soil layer was removed, the main nest of *T.sinensis* sp. nov. became visible (Fig. 4A).

Based on observed facts, the two species share an ant canal and inhabit the same nest area; however, *T.sinensis* sp. nov. builds its own nest and broods its eggs, leading to the hypothesis that *T.sinensis* sp. nov. may exhibit parabiosis in the nest of *D.rugosum*. This assumption is primarily supported by the significant body type and population of *D.rugosum*, which suggests that *T.sinensis* sp. nov. is unlikely to provide sufficient food for *D.rugosum*. Therefore, we preliminarily believe that *T.sinensis* sp. nov. may feed on the food scraps left by *D.rugosum* and share the foraging trails. However, the method or pathway by which *T.sinensis* sp. nov. enters the nest of *D.rugosum* remains unclear, and long-term observation is needed to uncover this mystery.

##### Recognition.

*Tetramoriumsinensis* sp. nov. bears a resemblance to *T.jarawa* (Agavekar, Hita Garcia & Economo, 2017) due to the presence of similar longitudinal striae in the frontal area and posterior part of the head slightly reticulate in the full-face view of the head. However, it can be distinguished from the latter by the lateral sides of the mesosoma exhibiting transverse striae (while entirely reticulate punctate in *T.jarawa*), the propodeal spines short triangular, as long as broad basally and the tip straight (while the propodeal spines are long, significantly longer than broad basally and the tip upturned in *T.jarawa*). In dorsal view, the petiole of *T.sinensis* sp. nov. is as long as broad, distinguishing it from *T.jarawa* where the petiole is longer than broad.

## ﻿Discussion

*Tetramoriumsinensis* sp. nov. is found in Fenghuang Mountain Park in Guangdong Province, China. Similar to the type localities of the *T.inglebyi*-group species, this new species is situated within the Oriental Region (Holt et al. 2013). This indicates that the group extends significantly further east, beyond the confines of the Indian Subcontinent, and there remains a wealth of undiscovered species yet to be explored.

Parabiosis refers to the phenomenon where two (or more) different ant species use the same nest while keeping their broods separate (Jeanne 2021). Although this type of interaction is uncommon, a few case studies have been published. For example, *Strumigenys* (Smith, 1860) and *Diacamma* (Mayr, 1862) were found living together in a compound nest and they have a significant size difference (Kaufmann et al. 2003). Considering the distinguishing features mentioned above, the noticeable difference in body size between *T.sinensis* sp. nov. and *D.rugosum* suggests that the new species is significantly smaller than *D.rugosum*. As well as the two species inhabiting the same nest area, there is only one tunnel, and in contrast to most other species of the genus, *T.sinensis* sp. nov. has a marked vestigial eye. Therefore, our preliminary findings suggest that it is consistent with parabiotic characteristics (Vantaux et al. 2007; Emery and Tsutsui 2016). However, whether there is a parasitic relationship or some kind of ecological interaction between them and how the queen of *T.sinensis* sp. nov. infiltrates the host nest remains uncertain, which warrants detailed observation to unravel this question.

### ﻿Key to members of the *Tetramoriuminglebyi*-group species based on the worker castes

**Table:** 

1	Maximum diameter of the eyes longer than maximum diameter of the antennal scapes (Fig. 5A)	***T.inglebyi***
–	Maximum diameter of the eyes shorter than maximum diameter of the antennal scapes	**2**
2	In dorsal view, petiole broader than long (Fig. 8C)	***T.elisabethae***
–	In dorsal view, petiole longer than broad or as long as broad	**3**
3	Anterior margin of clypeus concave in the middle	**4**
–	Anterior margin of clypeus flat or slightly convex	**5**
4	In dorsal view, head reticulated rugose, petiole longer than broad (Fig. 6C)	***T.myops***
–	In dorsal view, head longitudinal rugose, petiole as long as broad (Fig. 7C)	***T.triangulatum***
5	In lateral view, mesosoma very reticulate-punctate (Fig. 9D); in dorsal view, petiole longer than broad (Fig. 9C)	***T.jarawa***
–	In lateral view, mesosoma with a dense transverse striation (Fig. 1D); in dorsal view, petiole as long as broad (Fig. 1C)	***T.sinensis* sp. nov.**

**Figure 5.**
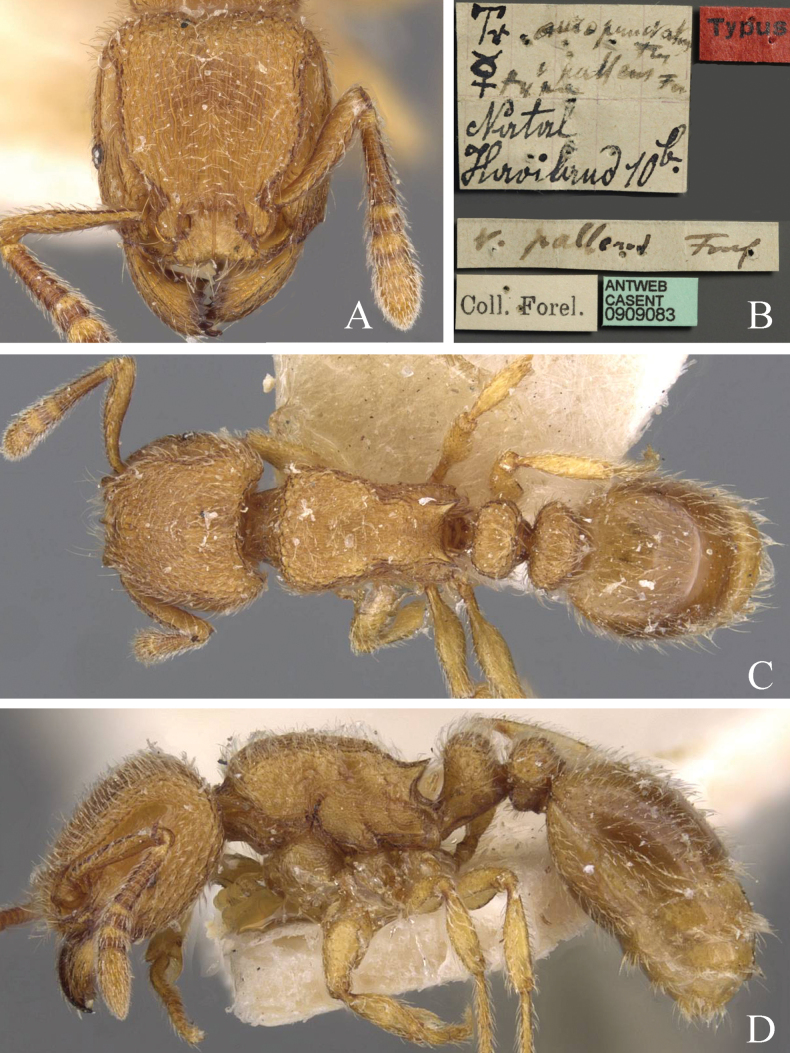
*Tetramoriuminglebyi*, worker. Head in full-face view (**A**), label of paratype (**B**), body in dorsal view (**C**), body in lateral view (**D**). Images sourced from AntWeb (2023) online at https://www.antweb.org.

**Figure 6.**
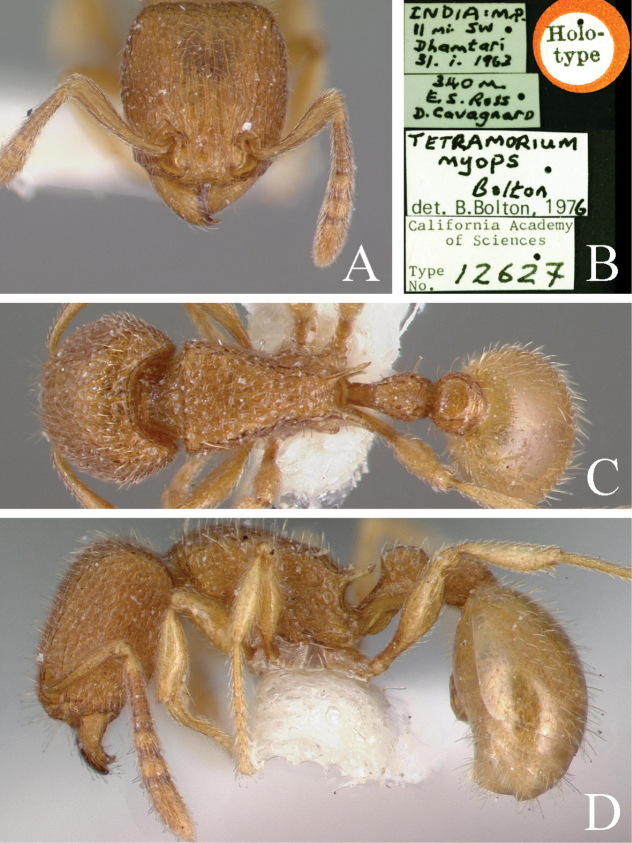
*Tetramoriummyops*, worker. Head in full-face view (**A**), label of paratype (**B**), body in dorsal view (**C**), body in lateral view (**D**). Images sourced from AntWeb (2023) online at https://www.antweb.org.

**Figure 7.**
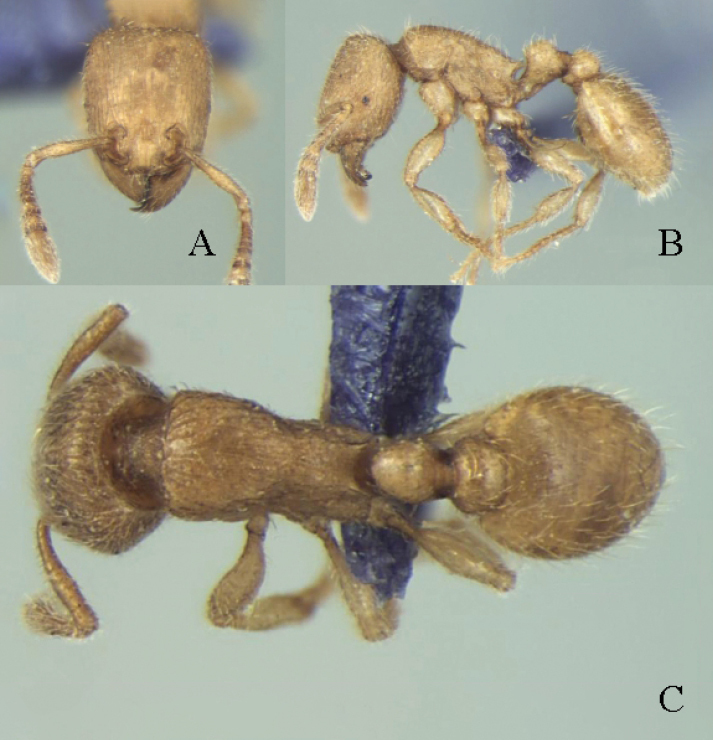
*Tetramoriumtriangulatum*, worker. Head in full-face view (**A**), body in lateral view (**B**), body in dorsal view (**C**). Images sourced from AntWiki (2023) online at https://www.antwiki.org.

**Figure 8.**
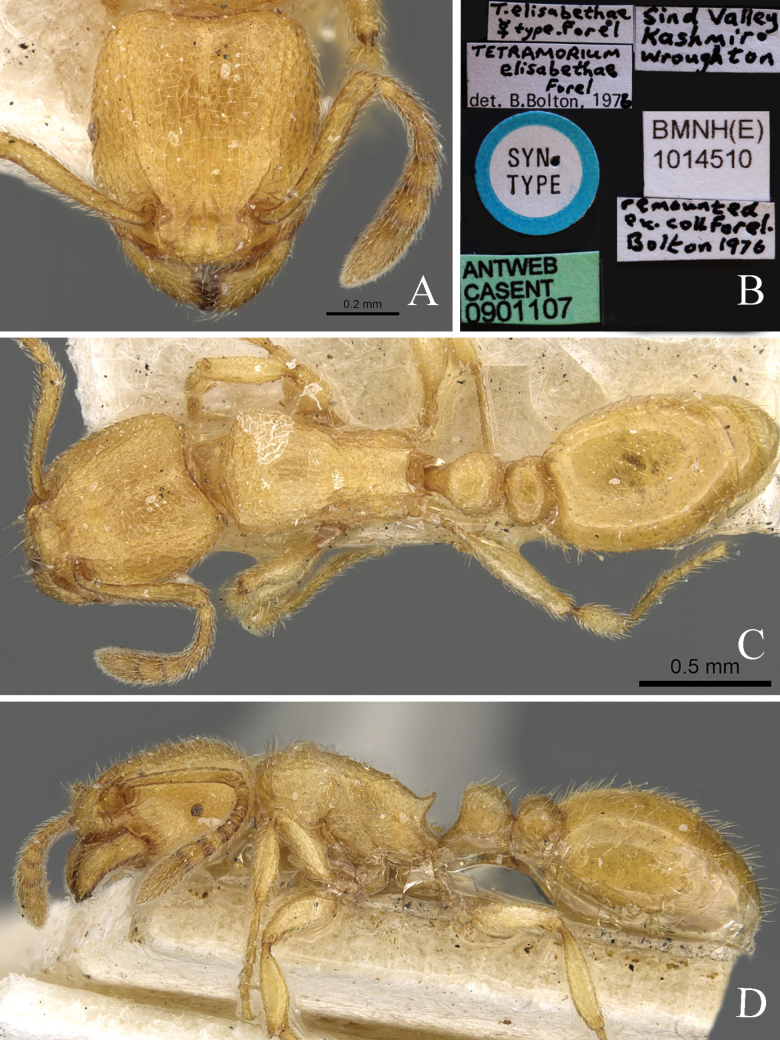
*Tetramoriumelisabethae*, worker. Head in full-face view (**A**), label of syntype (**B**), body in dorsal view (**C**), body in lateral view (**D**). Images sourced from AntWeb (2023) online at https://www.antweb.org.

**Figure 9.**
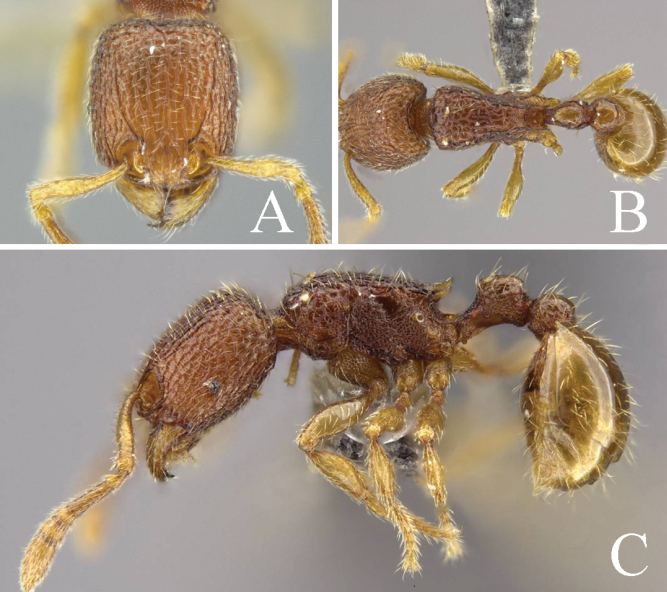
*Tetramoriumjarawa*, worker. Head in full-face view (**A**), body in dorsal view (**B**), body in lateral view (**C**). Images sourced from AntWeb (2023) online at https://www.antweb.org.

## Supplementary Material

XML Treatment for Tetramorium sinensis

## ﻿Additional information

### Author ORCIDs

Benan Zhang https://orcid.org/0000-0001-8427-7450

Congcong Du https://orcid.org/0000-0002-8078-4751

Zhilin Chen https://orcid.org/0000-0001-6564-1528

### Data availability

All of the data that support the findings of this study are available in the main text.
